# The Quality Characteristics of Black Soybean Compound Fermented Milk Using *Lactiplantibacillus plantarum* IMAU40001: A Physicochemical and Metabolomics Analysis

**DOI:** 10.3390/microorganisms14061209

**Published:** 2026-05-27

**Authors:** Liya Ai, Jie Zhang, Musu Zha, Yongfu Chen

**Affiliations:** 1 Inner Mongolia Key Laboratory of Dairy Biotechnology and Engineering, Ministry of Education, Inner Mongolia Agricultural University, Hohhot 010018, China; 2 Key Laboratory of Dairy Products Processing, Ministry of Agriculture and Rural Affairs, Hohhot 010018, China; 3 Inner Mongolia Key Laboratory of Dairy Biotechnology and Engineering, Inner Mongolia Agricultural University, Hohhot 010018, China

**Keywords:** *Lactiplantibacillus plantarum*, black soybean, quality evaluation, non-targeted metabolomics

## Abstract

This study investigated the effects of co-fermentation with *Lactiplantibacillus plantarum* IMAU40001 and the commercial starter PYS-010 on the properties of black soybean compound fermented milk (BSCFM). Specifically, the fermentation characteristics, viable bacterial counts, texture, sensory attributes, and metabolomic profiles of BSCFM were evaluated following co-fermentation. Notably, co-fermentation enhanced water-holding capacity and textural properties, while maintaining high probiotic viability (>10^6^ CFU/mL). Sensory evaluation indicated that co-fermentation improved the overall balance of texture in BSCFM. Non-targeted metabolomic analysis revealed an increased abundance of bioactive metabolites, including flavonoids and amino acids. KEGG pathway analysis indicated the enhanced metabolism of amino acids, lipids, and plant secondary metabolites. These results suggest that black soybean serves as a rich source of functional precursors that, upon co-fermentation, facilitate the biosynthesis of health-promoting compounds. Overall, co-fermentation with IMAU40001 and PYS-010 improves the structural, sensory, and functional properties of BSCFM, offering a promising strategy for the development of functional fermented dairy products.

## 1. Introduction

Due to increased focus on healthy and sustainable dietary habits among consumers, the interest in dairy alternatives has grown. In addition, concerns regarding animal welfare and environmental issues are also driving the development and consumption of plant-based fermented milks [1]. As a result, plant-based fermented products have emerged as a popular area of food research and innovation. Compared with conventional dairy fermented milks, plant-based fermented milks offer several advantages, including a broader range of raw material sources and reduced environmental impact, and they are suitable for lactose-intolerant and vegan individuals [2]. However, plant-based fermented milks also suffer from reduced flavor and texture. Therefore, we consider combining plant-based milk with cow’s milk to enhance health benefits while minimizing flavor loss. Protein content is another crucial factor, as it affects not only the nutritional quality of fermented milk but also its texture, water retention, and overall consumer acceptance. Therefore, developing plant-based compound fermented milks with superior sensory properties and higher protein content has become an important direction in fermented dairy product research. Black soybean, a nutritionally dense legume, is a good source of high-quality protein and functional bioactive components. It is particularly rich in anthocyanins, isoflavones, and polyphenols, thus demonstrating strong antioxidant capacity and significant potential for functional food applications [3]. Interestingly, a previous study by Kalkan et al. [4] reported that incorporating hazelnut milk into milk for probiotic fermented milk production can improve the product’s structure and texture, while also enhancing microbial activity and increasing the concentration of bioactive compounds. Similarly, incorporating black soybean into compound fermented milk formulations could enhance both the nutritional and functional properties of the product while also providing new opportunities for the development of innovative compound fermented milk products.

*Lactiplantibacillus plantarum* (*L. plantarum*) is an important member of the lactic acid bacteria (LAB) family. This Gram-positive microbe is widely distributed in fermented foods and plant-based substrates. It exhibits strong tolerance to acidic conditions and bile salts, as well as effective intestinal adhesion [5]. It plays a crucial role in maintaining gut microbial homeostasis and promoting host health, demonstrating significant probiotic potential [6]. Duan et al. [7] reported that *L. plantarum* can enhance host health through multiple mechanisms, including strengthening the gastrointestinal epithelial barrier, modulating immune responses, and contributing to the prevention of chronic metabolic diseases. Commercial starter cultures provide key advantages in terms of acidification rates, fermentation stability, and gel formation, thereby ensuring the physicochemical quality and processing consistency of fermented milk [8]. Co-fermentation with *L. plantarum* and commercial starter cultures enables the production of fermented milks with improved functional properties. Here, the interactions between bacterial strains can improve fermentation speed, enhance sensory quality, and promote the production of bioactive compounds [9], thereby improving functional characteristics compared to single-strain fermentation. For instance, a previous study demonstrated that co-fermentation with *Lactobacillus helveticus* H11 and commercial starter cultures reduced fermentation time while improving the quality and storage stability of fermented milk [10]. Furthermore, combining *L. plantarum* with commercial starters was found to improve the water-holding capacity, viscosity, hardness, and consistency of fermented milk and promote the accumulation of beneficial non-volatile metabolites, improving flavor profiles [8,11].

In recent years, non-targeted metabolomics has emerged as a powerful analytical tool for studying the changes in small-molecule metabolites in fermented dairy products, providing valuable insights into the biochemical properties of probiotic fermented milks [12]. However, systematic studies on the production of plant-based compound fermented milks via co-fermentation with probiotics and commercial starter cultures remain limited. Therefore, exploring the application and advantages of multi-strain co-fermentation strategies in plant-based compound fermented milk systems could provide valuable insights and drive the development of functional plant-based fermented milks.

*L. plantarum* IMAU40001—previously isolated by our research group from fermented mare’s milk in Qinghai Province, China—is a probiotic strain that shows strong tolerance to bile salts and simulated gastrointestinal conditions. It can also secrete stable antibacterial substances and has good antioxidant capacity, thus having a potential protective effect on intestinal health. Meanwhile, the commercial starter PYS-010 is widely used in industrial fermented milk production. This study aims to apply the newly discovered probiotic strain *L. plantarum* IMAU40001 and the commercial starter culture PYS-010 to the preparation of BSCFM. It is hoped that by combining plant-based and milk-based products with multi-strain co-fermentation, the formation of the gel network and textural properties of fermented milk can be synergistically improved. It is also hoped that the bioactive substances in the compound fermented milk can be increased and the functional properties can be enhanced. The objective was to systematically evaluate the quality characteristics of black soybean compound fermented milk (BSCFM) produced through co-fermentation with a combination of *L. plantarum* IMAU40001 and a commercial starter culture. This study focused on physicochemical properties and non-targeted metabolomic profiling. Specifically, key parameters such as fermentation time, pH, titratable acidity (TA), viable cell count, texture, and water-holding capacity (WHC) were examined, along with non-volatile metabolites. This study is expected to provide foundational data and theoretical support for the development of fermented dairy products.

## 2. Materials and Methods

### 2.1. The Bacterial Strains

The *L. plantarum* IMAU40001 (LP, IMAU40001) strain used in this study was obtained from the Lactic Acid Bacteria Culture Collection (LABCC) at Inner Mongolia Agricultural University. IMAU40001 was initially isolated from traditional fermented mare’s milk produced in Saishike Township, Wulan County, Haixi Prefecture, Qinghai Province. The commercial starter culture PYS-010 (CS), supplied by Beijing Ketuo Hengtong Biotechnology Co., Ltd. (Beijing, China), was composed of *Lactobacillus delbrueckii* subsp. *bulgaricus* and *Streptococcus thermophilus* (*S. thermophilus*).

### 2.2. Preparation of Fermented Milk

Black soybean milk and milk were mixed thoroughly (50%, *v*/*v*). The mixture was heated to 65 °C in a water bath and stirred for 15 min. Then, 6.5% (*w*/*w*) of white sugar (Guangxi FengTang Co., Ltd., Liuzhou, China) was gradually added under continuous stirring. The mixture was homogenized using an SHR homogenizer (Shenlu, Shanghai, China) at 65 °C and 20 MPa. Subsequently, the mixture was pasteurized at 95°C for 30 min. After cooling to 37 °C, the samples were divided into four groups, each characterized by different combinations of fermentation substrates and starter cultures: (1) group A, black soybean milk and milk co-fermented using IMAU40001 and PYS-010; (2) group B, black soybean milk and milk fermented with IMAU40001; (3) group C, black soybean milk and milk fermented with PYS-010; (4) group D, milk co-fermented with IMAU40001 and PYS-010.

The inoculated milk samples were placed in a 250 mL container and fermented at 37 °C until the pH reached 4.6–4.5. After fermentation, the samples were placed in a 4 °C refrigerator for about 12 h (post-fermentation), and relevant indicators were subsequently assessed.

### 2.3. Physicochemical Properties of Fermented Milk

#### 2.3.1. Determination of Fermentation Time, TA, and Viable Bacterial Counts

**pH:** The pH of the samples was measured using a pH meter (pHSJ-3F; LeiCi, Shanghai, China) until the fermentation endpoint was reached, and the fermentation time was recorded accordingly.

**TA:** Briefly, 10 g of fermented milk was mixed with 20 mL of cooled distilled water. Then, two drops of phenolphthalein indicator were added, and the mixture was shaken well. The fermented milk sample was titrated against a calibrated 0.1 mol/L NaOH solution (analytical grade; Tianjin New Technology Industrial Park Kemao Chemical Reagent Co., Ltd., Tianjin, China) until it turned slightly red and remained this color for 30 s [13].

**Viable bacterial count:** The fermented milk sample (25 mL) was diluted with 225 mL of sterile physiological saline and shaken for 15 min. Then, 1 mL of the sample was transferred to a tube containing 9 mL of physiological saline and serially diluted. Three optimal gradients were selected, and 1 mL of each gradient mixture was transferred to a sterile Petri dish. Solid MRS culture medium was then poured into the Petri dish [14]. The plates were incubated at 37 °C for 48 h under anaerobic conditions. The viable bacterial count was then recorded. All counts were performed in triplicate for each dilution, and results are expressed as log CFU/mL of fermented milk.

#### 2.3.2. Determination of Viscosity, Texture, and WHC

**Viscosity:** The viscosity of the fermented milk samples was determined using a Brookfield DV-1 viscometer (AMETEK Brookfield, Middleborough, MA, USA). Before analysis, all samples were equilibrated to room temperature (approximately 25 °C) and examined using a No. 4 rotor. Each sample was examined three times [15].

**Texture:** The hardness and consistency of the fermented milk samples were determined using a texture analyzer equipped with an A/BE probe (TA.XT.plus, Stable Micro Systems Ltd., Godalming, UK). The front and rear speeds were 15 mm/s, and the test speed was 10 mm/s. The initial force applied was 20 g, and the test distance was 20 mm. The samples were compressed to 20% of their original height for 5 s, as described by Bai et al. [8], to simulate chewing.

**WHC:** A 20 g sample of fermented milk was placed in a funnel containing filter paper and left at room temperature for 120 min. The filtrate was then collected and weighed [16]. The WHC was determined using the following formula:

WHC (%) = [1 − (Filtrate weight [g]/Sample weight [g])] × 100%

### 2.4. Sensory Profile Analysis

Each group of fermented milk samples was shaken well and poured evenly into tasting cups. The sensory assessment panel consisted of 10 trained evaluators. The sensory evaluation form (Table 1) was modified based on the overview provided by Zhang et al. [17], with each group of fermented milk samples assessed for color, taste, aroma, and texture. To minimize cross-sample influence, the evaluators rinsed their mouths during the assessment. Although this sensory evaluation did not require ethics approval, all panel members were fully informed regarding the purpose and content of the test and provided informed consent.

### 2.5. Untargeted Metabolomics Analysis

**Sample preparation:** To determine the concentration of non-volatile compounds in BSCFM, different samples were prepared according to the method described by Zha et al. [18]. Samples stored at −80 °C were thawed and allowed to reach room temperature, and acetonitrile was added subsequently at a ratio of 1:3 (LC-MS grade; Thermo Fisher, Waltham, MA, USA). The mixture was centrifuged at 1000× *g* and 4 °C for 10 min to obtain protein precipitates. The supernatant was collected and diluted 1:3, allowed to stand at 4 °C for 2 h, and then centrifuged at 12,000× *g* and 4 °C for 5 min. The resulting supernatant was reconstituted in 40% acetonitrile, filtered through a microporous membrane (0.22 µm), and collected in a sample vial. To assess the stability and reproducibility of the analytical system and conditions, quality control (QC) samples were prepared by pooling equal volumes of all samples.

**Ultra-performance liquid chromatography–mass spectrometry (UPLC-MS):** Chromatographic analysis was performed using an AB SCIEX TripleTOF 6600+ system (AB SCIEX, Redwood City, CA, USA) equipped with an Acquity UPLC HSS T18 column (1.8 µm; 2.1 mm × 100 mm). The column temperature was maintained at 35 °C. The flow rate was set to 0.35 mL/min, while the injection volume was 2.0 µL. Gradient elution was employed for sample separation. In the positive ion scan mode (ESI+), the mobile phase consisted of deionized water containing 0.1% formic acid (LC-MS grade; Thermo Fisher, Waltham, MA, USA) and acetonitrile (LCMS grade; Thermo Fisher, Waltham, MA, USA). In the negative ion scan mode (ESI−), the mobile phase consisted of deionized water containing 0.1% ammonium hydroxide (LCMS grade; Thermo Fisher, Waltham, MA, USA) and acetonitrile. The gradient elution programs for the two modes were as follows: 0 min, 98% A + 2% B; 0.25 min, 98% A + 2% B; 12 min, 2% A + 98% B; 14 min, 2% A + 98% B; 14.1 min, 98% A + 2% B; 17.0 min, 98% A + 2% B. The total runtime was 23 min.

### 2.6. Multivariate Statistical Analysis

All experiments were performed with three independent biological replicates (*n* = 3). For each biological replicate, three technical replicates were included. The average value of the three technical replicates was first calculated for each biological replicate, resulting in three independent data points per group (*n* = 3) for subsequent analysis. The Shapiro–Wilk test was used to assess the normality of the data, and the Levene test was used to assess the homogeneity of variance. Comparisons among multiple groups were performed using one-way ANOVA. When ANOVA indicated significant differences, Tukey’s honestly significant difference (HSD) post hoc test was applied to correct for multiple comparisons. Data on the physicochemical properties and target metabolites were assessed using analysis of variance with a 95% confidence level cutoff. Principal component analysis (PCA) and partial least squares discriminant analysis (PLS-DA) were employed to analyze the fermented milk metabolome. Significantly different metabolites were identified based on cutoff variable importance in projection (VIP) scores (VIP ≥ 1) and *p*-values (*p* < 0.05). Metabolic pathway annotation and differential metabolite enrichment analysis were performed using the online tool Metaboanalyst 5.0 (https://www.metaboanalyst.ca/, accessed on 18 March 2026). All statistical analyses and visualizations were performed using R (v4.3.1) and Origin 2025. Data are presented as mean ± standard deviation (SD), and differences were considered statistically significant at *p* < 0.05.

## 3. Results

### 3.1. Fermentation Characteristics of Fermented Milk

#### 3.1.1. Fermentation Time

**Figure 1:**
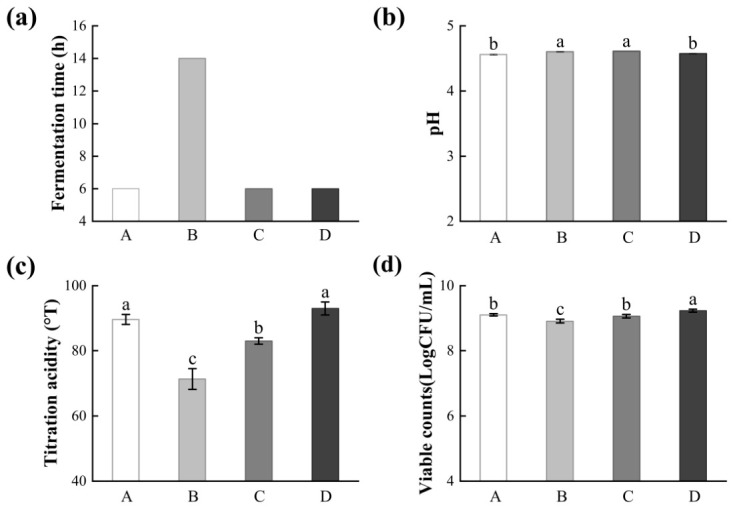
Fermentation time and physicochemical properties of fermented milk in every group. (**a**) Fermentation time; (**b**) pH; (**c**) Titratable acidity; (**d**) Viable counts. Different lowercase letters indicate significant differences in a certain parameter between different groups (*p* < 0.05). Group A, BSCFM co-fermentation with IMAU40001 and PYS-010; group B, BSCFM fermented with IMAU40001; group C, BSCFM fermented with PYS-010; group D, milk co-fermentation with IMAU40001 and PYS-010.

Fermentation time is a key indicator of the interaction between starter cultures and their substrates [10]. Moreover, in the fermentation industry, a longer fermentation time increases the product cycle and cost. Therefore, selecting an appropriate starter culture that shortens the fermentation time is extremely important. As shown in Figure 1a, the time taken to reach the fermentation endpoint (pH 4.6) varied among the different groups. Notably, the addition of PYS-010 significantly shortened the fermentation time. In the commercial starter culture PYS-010, *Lactobacillus delbrueckii* subsp. *bulgaricus* and *S. thermophilus* drive isoform fermentation to produce lactic acid [19]. Thus, efficient fermentation is achieved through the metabolic complementarity of these bacteria, synergistically enhancing the sensory and functional properties of fermented milk. Importantly, our findings showed that the addition of black soybean milk or IMAU40001 did not significantly alter the fermentation time. This indicated that changes in the substrate or fermentation strain did not significantly affect lactic acid production in this system.

#### 3.1.2. pH and TA

pH and TA are important indicators for evaluating the acidification of fermented milk and also affect the survival of LAB. Changes in acidity significantly impact the flavor and quality of fermented milk, with appropriate acidity levels improving both taste and quality. In this study, pH and TA values were found to differ across different fermented milk samples (Figure 1b,c). The pH values in groups B and C were significantly higher than those in groups A and D (*p* < 0.05); however, there was no significant difference in pH between groups A and D. Meanwhile, TA was significantly higher in groups A and D than in groups B and C (*p* < 0.05), and the final TA of group A was significantly higher than that of groups B and C (*p* < 0.05). This indicated that the acid-producing capacity of the single strain IMAU40001 in the milk matrix was limited, and co-fermentation with PYS-010 was more effective to achieve sufficient acidity. Moreover, there was no significant difference in the endpoint TA between group A and group D (*p* > 0.05), demonstrating that the introduction of black soybean milk as a substrate did not negatively impact the overall acid-production capacity of the compound fermentation system. Meanwhile, the final TA of group A was slightly lower than that of group D, consistent with the weaker buffering capacity of plant protein systems compared to that of casein systems. Notably, all four groups of samples showed pH values close to within the optimal pH range for drinking fermented milk (4.2–4.5) [20]. Similarly, all samples showed optimal TA values (70–110 °T) [21]. In particular, the endpoint TA of groups A and D was within the optimal range for fermented milk, indicating that the introduction of black soybean milk did not cause excessive acidification.

#### 3.1.3. Viable Bacterial Counts

The number of live bacteria is an important indicator of the probiotic properties and quality of fermented milk. As shown in Figure 1d, in all groups of fermented milk samples, the number of viable bacteria after fermentation was greater than 10^6^ CFU/mL, meeting the minimum functional requirements for probiotic products [22]. When the milk matrix contained black soybean milk, the number of viable bacteria in the fermented milk was significantly higher following co-fermentation with IMAU40001 and PYS-010 than following single-strain fermentation with IMAU40001 (*p* < 0.05). This indicated that PYS-010 may promote the proliferation of IMAU40001 through mutually beneficial metabolic activity, with co-fermentation producing a synergistic effect [23]. Such processes are known to typically enable the subsequent generation of active metabolites. Notably, when IMAU40001 was combined with PYS-010, the substrate significantly affected the viable bacterial count. In particular, the viable bacterial count in group A was significantly lower than that in group D. This lower count in group A could be attributed to the weaker buffering capacity of the plant protein system, leading to faster lactic acid accumulation, and the inhibition of LAB by anti-nutritional factors such as saponins and phytic acid in black soybeans [24]. Furthermore, the limited sources of carbon and available nitrogen in black soybean milk may have also hindered bacterial growth [25,26]. Despite synergistic effects, the final viable bacterial count was still lower in the compound substrate system than in the milk system. However, the viable bacterial count in group A still met the requirements for probiotic fermented milk and could still produce positive health-promoting benefits.

#### 3.1.4. WHC, Viscosity, and Texture

**Figure 2:**
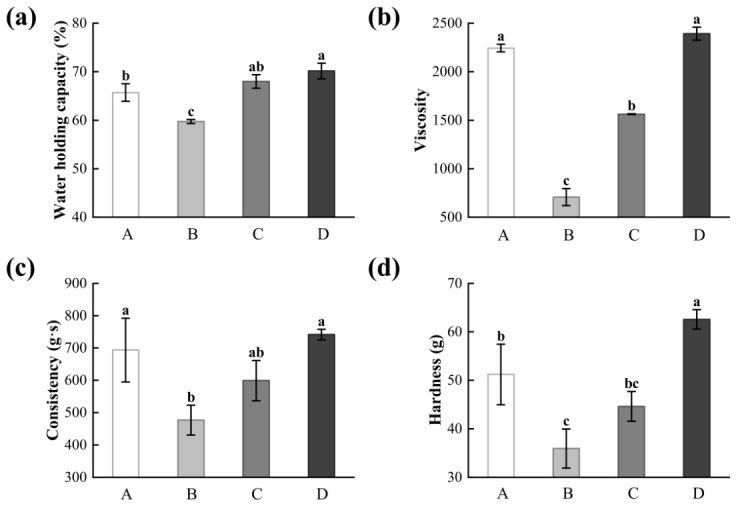
Water-holding capacity and textural properties of fermented milk in every group. (**a**) Water-holding capacity; (**b**) Viscosity; (**c**) Consistency; (**d**) Hardness. Different lowercase letters indicate significant differences in a certain parameter between different groups (*p* < 0.05).

WHC is an important criterion for evaluating the internal structural stability of fermented milk [27]. Among the black soybean–milk fermentation systems, the WHC of group A was significantly higher than that of groups B and C (Figure 2a). This was mainly due to two factors. First, the extracellular polysaccharides produced by IMAU40001 became embedded within the casein network, enhancing the three-dimensional structure of the gel and thus reducing whey precipitation [23]. Second, PYS-010 accelerated casein aggregation via robust acid production. Therefore, we hypothesize that the synergistic effect of these two factors helps to form a more compact protein network structure [10]. However, compared with group D, group A showed a slightly lower WHC. This was because black soybean protein replaced some of the casein, altering the dense and uniform gel network structure that was originally dominated by casein. The plant protein acted as a “filler” in the gel network instead of forming a continuous gel skeleton like casein [28]. Nevertheless, the WHC in group A was above 65%, which was sufficient. The addition of black soybean milk did not severely weaken the protein gel network structure, and the composite fermentation system helped maintain the WHC. However, current understanding of the protein structure is primarily inferred from WHC and textural properties. We acknowledge that direct structural characterization (e.g., scanning electron microscopy) is needed to confirm the proposed denser network, and this will be pursued in future studies.

In addition to WHC, viscosity and texture also serve as key parameters for evaluating the structural strength and physical properties of fermented milks [29]. The viscosity and texture of the different fermented milk samples (hardness and consistency) are described in Figure 2b–d. Overall, group D exhibited the highest viscosity, consistency, and hardness, which reflected the formation of a dense protein gel network due to the high casein content within the milk matrix. Although the addition of black soybean milk reduced the casein content in group A, the reduction was insufficient to significantly alter the gel structure. The exopolysaccharides (EPSs) produced after co-fermentation with IMAU40001 and PYS-010 were integrated into the protein framework [30], enhancing the three-dimensional gel network and improving textural properties [31]. In contrast, group B (single-strain fermentation) and group C (single commercial starter) showed significantly deteriorated textural properties (*p* < 0.05), with group B exhibiting the lowest viscosity, consistency, and hardness, potentially due to limited EPS production and weak acid-induced protein aggregation [32]. These results indicated that the synergistic effect of IMAU40001 and PYS-010 promotes the formation of a denser and more cohesive three-dimensional protein network in BSCFM, with EPS enhancing the protein scaffold, while PYS-010 effectively acidifies the matrix to induce protein aggregation [33]. Although the textural properties of group A were slightly inferior to those of group D, co-fermentation significantly improved the gel structure when compared to single-strain fermentation.

### 3.2. Sensory Characteristics of Fermented Milk

**Figure 3:**
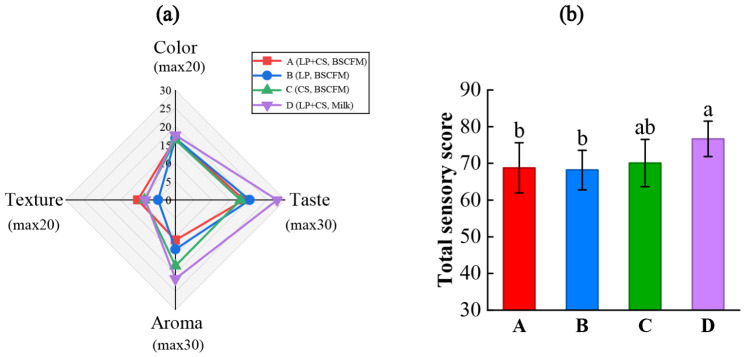
Sensory evaluation scores of the four groups. Sensory evaluation scores for each item (**a**); sensory evaluation total score (**b**). Different lowercase letters indicate significant differences in a certain parameter between different groups (*p* < 0.05).

The sensory evaluation of fermented milk is important as it reveals any differences in its quality and taste, and the popularity of the product can be assessed from a consumer’s perspective. As shown in Figure 3 and Supplementary Table S1, the total sensory score was the highest in group D, followed by group C, group A, and group B. While the addition of black soybeans lowered the sensory score of the fermented milk, it remained within an acceptable range (scores > 60), and co-fermentation slightly improved the sensory acceptance of BSCFM.

In terms of color, groups A and C acidified faster, resulting in more complete anthocyanin release and structural transformation, leading to a darker color and a slight decrease in color score. Group B acidified more slowly, with a color similar to group D. Overall, the scores of the four groups were quite close. Regarding taste, group D tasted the best, followed by group B, with groups A and C slightly lower. IMAU40001 may have a strong proteolytic ability, producing abundant free amino acids and organic acids, resulting in a mellow and refreshing flavor [34]. Co-fermentation and the addition of black soybeans may have affected the fermentation time and acid production of the strain, leading to increased acidity and a decrease in the score of group A, which is consistent with the previous TA results. In terms of aroma, group D scored the highest, and group A the lowest, mainly due to the beany smell of black soybeans. Regarding texture, group A scored the highest, attributed to the EPSs embedded in the protein network produced by IMAU40001, combined with the rapid acidification induced by PYS-010, which induced protein aggregation [29,35], forming a dense three-dimensional gel structure. Simultaneously, black bean protein and casein complemented each other through hydrogen bonds, disulfide bonds, and hydrophobic interactions, further enhancing the gel structure. In summary, although the BSCFM sensory score is slightly lower than that of regular fermented milk, it is still within an acceptable range. By employing a co-fermentation strategy, the texture was improved while maintaining a balance of aroma and flavor, adding certain functional characteristics within a sensory acceptable range, thus providing a feasible path for developing functional plant–milk blended fermented milks.

### 3.3. Metabolic Characteristics of Fermented Milk

#### 3.3.1. Metabolic Profiles

**Figure 4:**
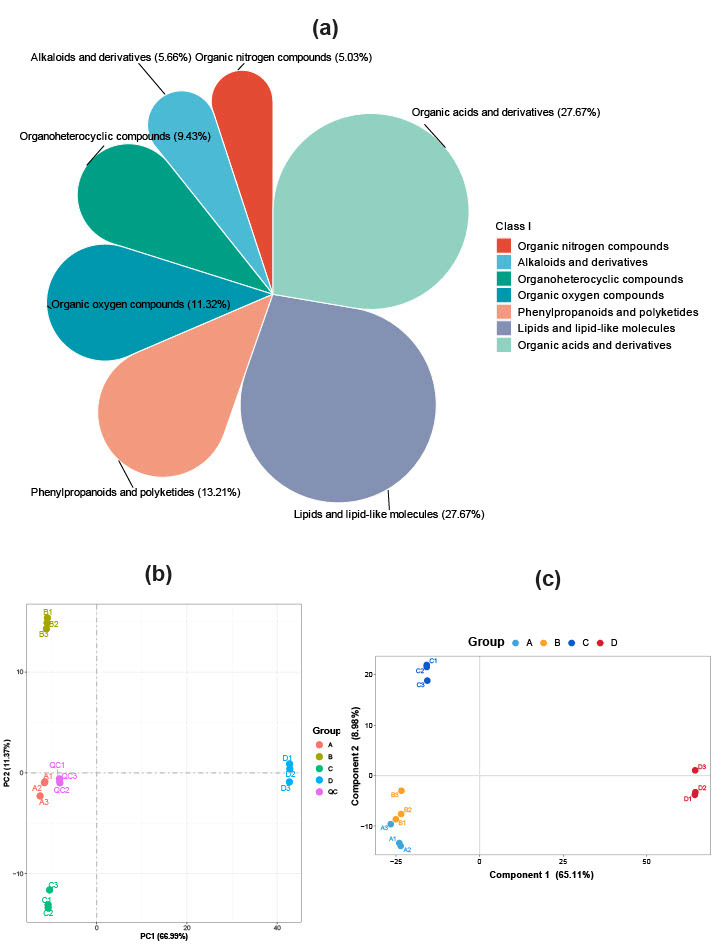
The overview of metabolites in fermented milk samples. The percentage of primary metabolites by category in all samples (**a**); PCA (**b**); PLS-DA (**c**). QC represents quality control samples.

To further elucidate the characteristic chemical components and metabolic pathways involved in fermented milk production, the matured samples were analyzed using non-targeted metabolomics. Under both the positive ion (ESI+) and negative ion (ESI−) modes, a total of 3311 metabolite ion features were detected, of which 159 metabolites (approximately 4.8%) were successfully annotated (Table S2, Figure 4a). This limited coverage is due to the stringent identification criteria, conservative annotation strategies, and database limitations; subsequent analyses are primarily based on these annotated metabolites. The primary classification of the annotated metabolites included 44 lipids and lipid-like molecules, 44 organic acids and their derivatives, and 21 phenylpropanoids and polyketides, encompassing a total of seven categories. The secondary classification further revealed 39 carboxylic acids and derivatives, 19 prenol lipids, and 18 organic oxygen compounds, covering 34 categories. These results provided a preliminary overview of the metabolic profile of BSCFM.

PCA, a widely used unsupervised dimensionality reduction technique in metabolomics, was applied to reveal clustering patterns and intergroup differences among samples [36]. PCA reduces data dimensionality through linear transformation. As shown in Figure 4b, the cumulative explained variance among the samples reached 78.36%, with the first principal component (PC1) contributing 66.99% and PC2 contributing 11.37%. Each scatter point in the PCA plot represents an individual sample, and samples with similar metabolic profiles are clustered closely together. By contrast, those with distinct profiles are more widely separated. In this study, the samples from the four groups exhibited clear separation, with group D showing the most distinct clustering relative to the other groups. Within-group clustering was tight, indicating good reproducibility and suggesting that both the starter cultures and the milk matrix significantly affect metabolite generation. QC samples, used to assess the repeatability of the analysis, formed a tight cluster, demonstrating high instrument precision, repeatability, and stability in metabolite detection. The QC samples were also clearly separated from the experimental samples. Subsequently, PLS-DA, a supervised multivariate statistical technique, was employed to further explore the differences in metabolite profiles among the groups (Figure 4c). The contribution rates of component 1 and component 2 were 65.11% and 8.98%, respectively. The PLS-DA score plot also revealed clear separation among the four groups along both components, reflecting their distinct metabolic profiles. No significant outliers were observed, further highlighting the inherent metabolic differences among the groups.

#### 3.3.2. Analysis of Differentially Abundant Metabolites

**Figure 5:**
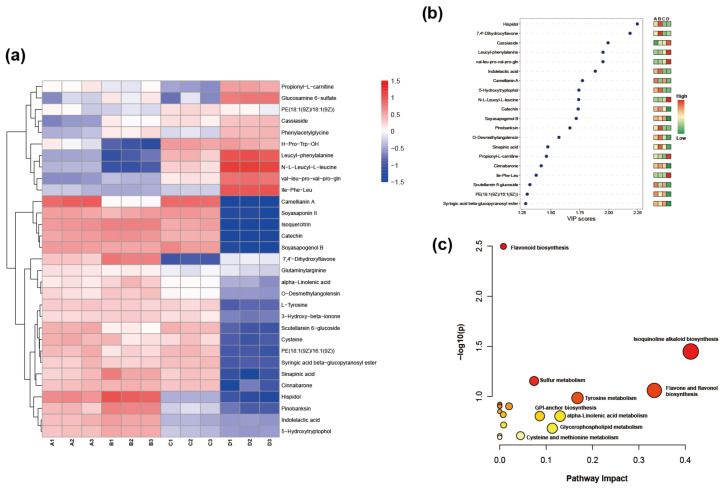
Comparison of metabolic profiles in different samples. The heatmap shows hierarchical clustering analysis (HCA) of metabolite changes in each group (**a**); analysis based on PLS-DA, considering the importance of projected variables (VIP) (**b**); KEGG pathway analysis of differentially expressed metabolites (**c**).

Differential metabolites were screened based on VIP values (VIP ≥ 1) and statistical significance (*p* < 0.05). Following annotation using the Human Metabolome Database (HMDB), the LIPID MAPS Structure Database (LMSD), and the relevant literature, a total of 31 differentially expressed metabolites were identified and subjected to hierarchical clustering analysis (HCA) (Figure 5a). These metabolites were primarily classified as phenylpropanoids and polyketides, organic acids and their derivatives, and lipids and lipid-like molecules. The HCA heatmap revealed the clear differentiation of metabolite profiles between groups A and D. In contrast, differences between groups A and B, as well as between groups A and C, were less pronounced. This pattern was consistent with the clustering trends observed in the PCA and PLS-DA plots.

Subsequently, the differential metabolites among the different groups were further analyzed (Figure 5b, Table S2). Compared with group B, group A exhibited higher peak intensities primarily for organic acids and their derivatives, lipids and lipid-like molecules, and phenylpropanoids and polyketides. In comparison with group C, group A also showed larger peak areas for organic heterocyclic compounds and organic oxygen-containing compounds, in addition to the three aforementioned categories. According to references, these metabolite classes are generally associated with potential health benefits, such as antioxidant and anti-inflammatory properties. Thus, co-fermentation with IMAU40001 and PYS-010 may have facilitated the accumulation of these metabolites through mechanisms such as substrate cross-utilization and inter-strain metabolic interactions. Among the identified metabolites, L-tyrosine, cysteine, and glutaminyl–arginine specifically belong to the class of carboxylic acids and their derivatives. During fermentation, LAB exhibit active metabolism, particularly amino acid biosynthesis and transformation. Multi-strain co-fermentation thus appeared to be more conducive to the production of beneficial metabolites in BSCFM. LAB fermentation promotes protein degradation and releases a variety of amino acids, among which cysteine is an aminothiol containing a thiol group and has a strong free radical scavenging ability [37]. Glutaminyl–arginine (Glu–Arg) exhibits potent lipase-inhibitory effects and functions as an enzyme regulator [38]. The fermented milk rich in these metabolites may have potential applications as a functional food for metabolic regulation. In addition, two other flavonoids (camellianin A and scutellarein 6-glucoside) were found to accumulate at high levels in BSCFM. Phosphatidylethanolamine species such as PE (18:1(9Z)/16:1(9Z)), a glycerophospholipid, may enhance lipid digestion and absorption and improve energy metabolism by modulating membrane permeability and may also function as potential emulsifiers [39]. Among these seven metabolites, L-tyrosine, cysteine, propionyl-carnitine, and glutaminyl–arginine were present at lower levels in group C. This indicated that commercial starter cultures alone may have limited capacity to produce certain functional metabolites in fermented milk. In contrast, the inclusion of IMAU40001 could enhance their accumulation. We speculate that multi-strain co-fermentation may help promote synergistic pathways such as proteolysis and amino acid metabolism, thereby increasing metabolite yield.

In addition to the seven metabolites described above, eight other compounds, including soyasaponin II and soyasapogenol B, were significantly more abundant in group A than in group B. Meanwhile, twelve other metabolites, including catechin and hispidol, were significantly more abundant in group A than in group C. The metabolites showing higher levels relative to group B were mainly prenol lipids and carboxylic acids and their derivatives, primarily obtained from soybean saponins and their aglycones. In addition, peptides and amino acids were derived via protein hydrolysis, and lipids and phenolic metabolites were also detected. Soyasaponin II and soyasapogenol B, which are abundant in soybean seeds, have been reported to possess significant bioactivities. However, these compounds may also contribute to undesirable flavors in soy milk. Therefore, balancing its functional activity and sensory quality is also worth noting. Additionally, the higher levels of PE (18:1(9Z)/18:1(9Z)) in milk systems may help enhance its antioxidant capacity [40].

Compared with group C, the significantly elevated metabolites in group A were primarily flavonoids and indoles and their derivatives, whose production and transformation appeared to depend on the presence of IMAU40001. Both indolelactic acid (ILA) and 5-hydroxytryptophol are involved in tryptophan metabolism; ILA is a microbial catabolite produced by LAB, while 5-hydroxytryptophol has demonstrated anti-angiogenic, antiproliferative, and pro-apoptotic effects on endothelial and tumor cells [41]. In this study, these functionally significant metabolites were notably enriched in group A. Therefore, we hypothesize that the metabolic potential of *L. plantarum* appears to be fully utilized in the co-fermentation system, complementing the activity of commercial starter cultures, thereby enabling broader biotransformation of substrates.

Black soybeans are rich in protein, exhibit favorable processing properties, and possess diverse biological activities. Fermentation further enhances their value by promoting the formation of beneficial metabolites. To elucidate the contribution of black soybean milk to BSCFM, the metabolite differences between groups A and D were compared. Relative to group D, group A showed significant enrichment for multiple metabolite classes, including flavonoids, carboxylic acids and their derivatives, and prenol lipids. The incorporation of black soybean milk as a fermentation substrate introduced bioactive plant constituents, such as flavonoids and saponins, which were further converted and released by LAB during co-fermentation, highlighting the unique functional advantages of black soybean milk as a fermentation substrate. For instance, hispidol and camellianin A were present at substantially higher levels in group A than in group D. These compounds, commonly derived from plant sources, exhibit a range of physiological activities. Notably, hispidol has been isolated from soybeans and demonstrates antibacterial and antioxidant properties [42]. Meanwhile, O-desmethylangolensin is produced by LAB, which exhibits high bioavailability and strong antioxidant activities [43]. In addition, black soybeans are rich in phenolic acids, which are released during fermentation through the action of microbial glycosidases and esterases. This may help to impart functional activity to fermented milk. Sinapinic acid has free radical-scavenging capacity and the ability to reduce intracellular reactive oxygen species (ROS) levels [44]. Furthermore, α-linolenic acid and 3-hydroxy-β-ionone, which are widely distributed in plant systems, exhibit antioxidant, anti-inflammatory, and gut microbiota-regulating activities [45]. Their presence in the fermented milk samples could be attributed to the high content of unsaturated fatty acids and carotenoids in black soybeans. During co-fermentation, microbial enzymes such as lipases and carotenoid-cleaving enzymes can release or transform these lipid-active compounds, thereby enhancing the functional properties of BSCFM. In addition, metabolites such as glutaminyl–arginine, cysteine, and L-tyrosine are closely associated with the hydrolysis of black soybean proteins. Compared with milk proteins, black soybean proteins possess a distinct amino acid composition; thus, the incorporation of black soybean milk, combined with the synergistic effects of co-fermentation, may facilitate the release of a broader spectrum of bioactive amino acids and peptides. This study found that the co-fermentation of black soybeans and milk enhanced the metabolic interactions and functional synergies between plant and animal components. The levels of various beneficial metabolites in group A were increased. Co-fermented BSCFM shows enhanced metabolite diversity and potential functional attributes, which warrant further in vitro and in vivo validation. It should be noted that the present study did not directly assess the biological activity, concentration, or bioavailability of these metabolites; therefore, the reported functional associations are based on the previous literature and should be interpreted with caution.

#### 3.3.3. KEGG Metabolic Pathway Enrichment

**Figure 6:**
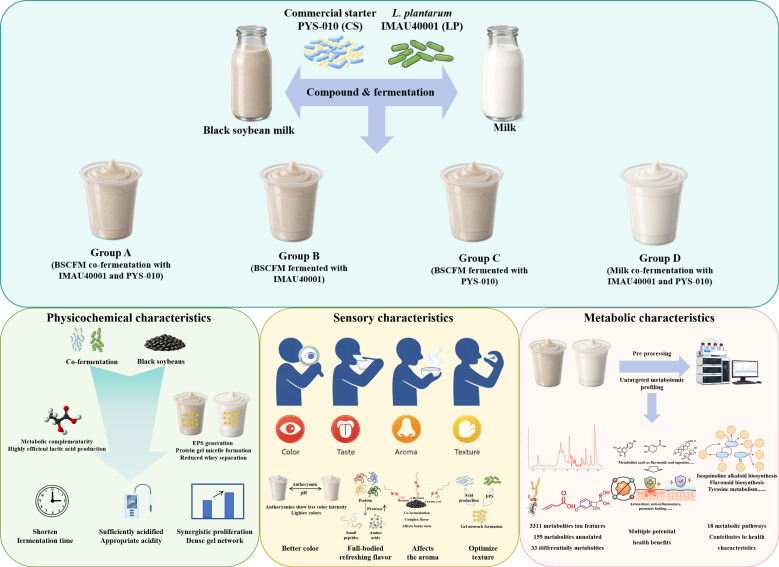
Overview of physicochemical, sensory, and metabolic characteristics of black soybean compound fermented milk.

As shown in Figure 5c, KEGG metabolic pathway enrichment analysis was conducted to explore the biochemical pathways associated with differentially abundant metabolites among the four sample groups [46]. In total, 18 different metabolic pathways were found to be enriched, including isoquinoline alkaloid biosynthesis, flavonoid biosynthesis, sulfur metabolism, flavonone and flavonol biosynthesis, and tyrosine metabolism. These pathways were primarily related to plant secondary metabolism, the metabolism of sulfur- and nitrogen-containing compounds, cofactor synthesis, and lipid metabolism. Additionally, they were closely associated with the functional properties of the fermented milk samples. Notably, three differentially abundant metabolites were enriched in the flavonoid biosynthesis pathway, which is among the core pathways of plant secondary metabolism: pinokansin, catechin, and 7,4′-dihydroxyflavone [47]. This suggested that the co-fermentation system may facilitate the metabolic transformation of the flavonoids found in black soybeans, producing flavonoids or flavonoid derivatives that have antioxidant and anti-inflammatory functions, and thus having a positive impact on the health properties of the product. Notably, L-tyrosine was enriched in both the isoquinoline alkaloid biosynthesis and tyrosine metabolism pathways. The enrichment of these pathways reflected a high rate of black soybean and milk protein hydrolysis in the co-fermentation system. In addition to acting as a nutrient, L-tyrosine also acts as a precursor for antioxidant production. Thus, elevated levels of this compound may enable ROS scavenging [48]. This helps increase the content of bioactive compounds in fermented milk. Isoquercitrin, enriched in the flavone and flavonol biosynthesis pathway, represents the glycoside form of quercetin. In this study, the co-fermentation process likely enhanced isoquercitrin conversion by promoting LAB metabolism. Meanwhile, the enrichment of the α-linolenic acid metabolism pathway reflected the unique lipid contribution of black soybeans as a plant-based raw material [6]. Co-fermentation appeared to promote the release or stabilization of α-linolenic acid. When IMAU40001 and PYS-010 were used for co-fermentation, they synergistically activated the metabolism of amino acids, lipids, and plant secondary metabolites. This is significant for the conversion of nutrients in black soybean milk substrate into active metabolites, and may further endow fermented milk with antioxidant, anti-inflammatory, and other physiological activities. Co-fermentation and the addition of black soybeans bring many potential benefits to fermented milk, which also lays the foundation for the application of black soybean milk. (Figure 6). However, a substantial number of potentially beneficial metabolites remain unidentified, and the functional roles of the annotated metabolites in plant-based fermented milk warrant further investigation. In addition, the interaction mechanisms between LAB such as *L. plantarum* and commercial starter cultures also need to be elucidated further to improve the quality and functional properties of fermented milk products and enable the targeted development of plant-based fermented dairy products with functional characteristics. Future research should integrate emerging technologies such as genomics, transcriptomics, and proteomics to provide deeper insights into the mechanisms underlying probiotic–starter co-fermentation and to support the targeted development of functional fermented dairy products.

## 4. Conclusions

In this study, co-fermentation with IMAU40001 and PYS-010 effectively improved the fermentation performance and overall quality of BSCFM. This combination may improve WHC by promoting the formation of a denser gel network, thus giving the product superior texture properties compared with single-strain fermentation, while maintaining high viable bacterial cell counts. Sensory evaluation indicated that the co-fermentation of IMAU40001 and PYS-010 in BSCFM produced the highest texture scores, while maintaining acceptable flavor and aroma. Metabolomic analysis further revealed that co-fermentation increased the abundance of bioactive compounds such as L-tyrosine, cysteine, and glutaminyl–arginine, which contain bioactive substances. Finally, KEGG pathway analysis suggested that the metabolism of amino acids, lipids, and plant secondary metabolites was enhanced after co-fermentation of the black soybean–milk substrate, reflecting the synergistic interactions between the starter cultures and substrate components. Overall, the introduction of black soybeans as a fermentation substrate, combined with multi-strain co-fermentation, improved the functional and sensory properties of fermented milk. Therefore, this strategy could be a feasible approach to developing plant-based functional fermented dairy product alternatives with higher nutritional value and bioactivity potential. However, the actual consumer perception and acceptance of BSCFM compared to existing commercially available products have not yet been evaluated. Further research through sensory evaluation panels and market assessments is needed to further demonstrate the product’s acceptability and feasibility.

## Figures and Tables

**Table 1:** Rules for sensory scoring. The fermented milk is scored according to its color, taste and flavor, and texture state.

Sensory Item	Sensory Scoring Standards	Score
Color	The color is uniform, exhibiting a characteristic milky white or light beige hue; the overall color is consistent.	15~20
	The color is slightly uneven, either too light or too dark; overall acceptable.	10~ 14
	The color is noticeably uneven or abnormal; appearance is poor.	0~9
Taste	The sweet and sour ratio is well-balanced and prominent, with a rich and mellow fermented milk flavor; the aftertaste is pleasant and harmonious.	20~30
	The sweet and sour balance is relatively good, with a passable fermented milk flavor; the aftertaste is slightly pleasant.	10~19
	Too sour or too sweet, or has an off-flavor; the aftertaste is unpleasant.	0~9
Aroma	No noticeable beany smell, no other odors; fresh and pleasant.	20~30
	Slight beany smell, no noticeable other odors; no noticeable unpleasant smells; acceptable.	10~19
	Noticeable beany smell, rancid smell, or other strong, unpleasant odors.	0~9
Texture	Fine texture, uniform consistency, no whey separation, moderate viscosity, smooth and delicate mouthfeel.	15~20
	Fair texture, with a small amount of whey separation, fairly delicate mouthfeel.	10~ 14
	More whey separation, coarser mouthfeel.	0~9

## Data Availability

Data will be made available on request.

## Supplementary Materials

The following supporting information can be downloaded at: https://www.mdpi.com/article/10.3390/microorganisms14061209/s1, Table S1: Sensory evaluation scores for four group; Table S2: Metabolites results of four groups.

## Abbreviations

The following abbreviations are used in this manuscript:

BSCFM	black soybean compound fermented milk
TA	titratable acidity
WHC	water-holding capacity
LABCC	Lactic Acid Bacteria Culture Collection
QC	quality control
PCA	principal component analysis
PLS-DA	partial least squares discriminant analysis
VIP	importance of projected variables
